# Population divergence in nutrient-temperature interactions in *Pieris rapae*

**DOI:** 10.3389/finsc.2023.1237624

**Published:** 2023-09-11

**Authors:** Anna L. Parker, Joel G. Kingsolver

**Affiliations:** Department of Biology, University of North Carolina, Chapel Hill, NC, United States

**Keywords:** cabbage white butterfly, climate change, macronutrients, phenotypic plasticity, population differentiation, temperature

## Abstract

The interaction between larval host plant quality and temperature can influence the short-term physiological rates and life-history traits of insect herbivores. These factors can vary locally, resulting in local adaptation in responses to diet and temperature, but the comparison of these interactions between populations is infrequently carried out. In this study, we examine how the macronutrient ratio of an artificial diet determines the larval growth, development, and survival of larval *Pieris rapae* (Lepidoptera: Pieridae) at different temperatures between two invasive North American populations from different climatic regions. We conducted a fully factorial experiment with three temperature treatments (18°C, 25°C, and 32°C) and three artificial diet treatments varying in terms of the ratio of protein to carbohydrate (low protein, balanced, and high protein). The effects of diet on life-history traits were greater at lower temperatures, but these differed between populations. Larvae from the subtropical population had reduced survival to pupation on the low-protein diet in the cold temperature treatment, whereas larval survival for the temperate population was equally high for all temperature and diet treatments. Overall, both populations performed more poorly (i.e., they showed slower rates of consumption, growth, and development, and had a smaller pupal mass) in the diet with the low protein ratio, but larvae from the temperate population were less sensitive to diet ratio changes at all temperatures. Our results confirm that the physiological and life-history consequences of imbalanced nutrition for insect herbivores may depend on developmental temperatures, and that different geographic populations of *P. rapae* within North America vary in their sensitivity to nutritional balance and temperature.

## 1 Introduction

The nutritional value of host plants has profound effects on the ecology and physiology of herbivores and is determined by multiple traits, including the quantity and quality of various nutrients, water content, and secondary defensive chemistry composition (1, 2). Macronutrient quantity and the quality of host plants can change among species and populations of the same species due to variations in the soil type, water stress, and local nitrogen deposition patterns (3, 4). Different parts of the same individual plant have also been shown to vary in macronutrient content (5). Two important macronutrients—dietary protein and carbohydrate—have been studied most extensively in herbivore nutritional ecology (6, 7). The balance of these two macronutrients influences insect herbivore performance and food selection (reviewed in 6).

For many insect herbivores, the uptake of protein and carbohydrates has the greatest influence on growth and body mass. Protein is a source of nitrogen for the growth and maintenance of tissues, the production of enzymes, and is a source of metabolic energy. Carbohydrate is a major source of metabolic energy and is also used for the production of body lipids and non-essential amino acids, and for structural purposes (i.e., cuticle deposition) (8). Experiments using artificial diets have shown that insects have the ability to adjust their feeding behavior and post-ingestion physiology to regulate the intake of these macronutrients (reviewed by 9, 10). For example, some insects can increase their consumption when fed nutritionally deficient foods (a behavior known as compensatory feeding) to achieve optimal intake targets of a specific macronutrient (11–15). Other insects are able to acclimate to nutritionally deficient diets and elongate their guts to increase the surface area over which nutrient absorption takes place and increase the efficiency of nutrient uptake (16–18). Therefore, macronutrient balance is a primary factor in determining the rates of development and growth in insect herbivores (13, 19, 20), including some Lepidoptera (6, 21).

Because temperature influences the metabolic rate and energy requirements of ectotherms, temperature, and nutrition together greatly influence the life-history outcomes for ectotherms (22–24). Numerous studies have documented differences in the thermal sensitivity and physiological rates and life-history traits of different populations and species (25). For example, studies with the invasive butterfly *Pieris rapae* in North America show that at intermediate (i.e., 20°C–27°C) rearing temperatures, larval development times varied among populations from different climatic regions (26–28). However, the ways in which geographic differences in life history vary across a wider temperature range and interact with nutrient quality are poorly understood.

Some studies have examined the effects of macronutrient ratio on thermal performance in insects, but most of this work has been conducted using Orthopterans, including locusts (10, 29, 30) and grasshoppers (17), which are mobile both as nymphs and as adults and therefore more able to make behavioral choices regarding nutrition and microclimate. In contrast, less work has been conducted on the less-mobile larval life stages in Lepidoptera and other holometabolous insects (21, 31, 32 for macronutrient ratio studies). Some recent work has begun characterizing optimal thermal and macronutrient conditions, investigating tradeoffs with different components of fitness (33). Additionally, many experimental studies have quantified how nutrition and temperature jointly affect short-term physiological rates, such as larval consumption, growth, and frass production (10, 34–37). Yet few studies quantify differences in life history in relation to changes in short-term physiological and development traits (38).

The present study uses artificial diets that vary in the macronutrient ratio of protein (P) to carbohydrate (C) to determine how nutrient ratio and rearing temperature combine to influence short-term physiological rates (larval consumption, frass production, and growth) and long-term life-history traits (survival, development time, and final body mass) in *P. rapae* (Lepidoptera: Pieridae). We address two main questions: (1) does rearing temperature alter the effects of the P:C ratio on the physiological rates and life-history traits of *P. rapae*? and (2) do these interactions differ between populations from differing climatic regions within North America? We address these questions using a full-factorial experiment with three temperature treatments and three artificial diet treatments varying in terms of the ratio of protein to carbohydrate (P:C).

## 2 Methods

### 2.1 Study system

We studied the reaction norms for larval life-history traits in two populations of the invasive cabbage white butterfly (*P. rapae*). *P. rapae* is native to Europe and utilizes wild and domesticated members of the Brassicacea family as larval host plants. It was introduced to south-eastern Canada on cabbages imported from Europe in the 1860s and rapidly colonized most of North America, spreading across the continent (39). We considered two climatically distinct populations from eastern North America: the first was near Chapel Hill, NC (USA). and the second was near Halifax, Nova Scotia (Canada) (hereby referred to as NC and NS, respectively). The NC population (36°N latitude) lives in a humid subtropical climate with an adult flight season from April to October and peak abundances in May–June and September–October; the mean daily maximum air temperatures during mid-summer are 31°C–32°C. The NS population (45°N latitude) lives in a temperate climate with a restricted adult flight season from June to August, peaking in July; the mean daily maximum air temperatures during mid-summer are 22°C–23°C. Previous studies showed that, at intermediate rearing temperatures (i.e., 20°C–27°C), these populations differ in terms of their mean development time and immune responses (26, 40, 41).

#### 2.1.1 Artificial diets

The diets used included a 1P:2.5C (low protein), 1P:1C (balanced), and 2.5P:1C (high protein) and all diets contained the same overall caloric content (**Table S1**). In this study, we define “balanced” in terms of a 1P:1C ratio; the “optimal” P:C ratio may differ from a 1P:1C ratio and may vary among different traits and species (13, 19). Following the method for a modified “high protein” lepidopteran diet in the study by Kingsolver and Woods (35), originally derived from that by Troetschler et al. (42), we prepared a dry, meridic diet that was successfully used to raise wild *P. rapae* in the lab (description in **Supplementary Material**, **Table S1**). The mixture of dry ingredients was blended with a 2% agar solution in a 6:1 agar solution to dry ingredient diet ratio. Collard powder, prepared by pulverizing dried, 12-week-old, greenhouse-grown collard greens (*B. oleraceae*) was added to act as a feeding stimulant for *P. rapae*. A subset of five diet block samples ranging between 1,500 mg and 2,000 mg of each diet type was lyophilized and analyzed for total N and C content using an elemental analyzer at the NCSU EATS laboratory in Raleigh, NC, USA (description in **Supplementary Material**).

### 2.2 Experimental design

In June and July of 2015, we developed a full-factorial design consisting of three artificial diets varying in macronutrient ratio (i.e., P:C) with three developmental temperatures (28°C, 25°C, and 32°C) on the NC population (**Table 1**). In August and September of 2015, we conducted the experiment on the NS population using the same full factorial design. Gravid females were collected from an organic farm in Cedar Grove, NC, USA (36°N), during June 2015 and brought back to laboratory facilities at the University of North Carolina at Chapel Hill, NC, USA. Eggs were obtained in a similar manner from adult females collected at an organic farm in Wolfville, Nova Scotia, Canada (45°N) in August 2015. These females were immobilized in glassine envelopes and shipped overnight to Chapel Hill, NC, USA (26). Our experiment included 21 families of *P. rapae* caterpillars from North Carolina, USA (N=191), and 17 families from Nova Scotia, Canada (N=325).

**Table 1:** Summary of experimental design and measurements.

	Pre treatment	Treatments	Measurements	
**Stage**	First ➔ through third	Fourth ➔ pupae	**Short-term rates during the 48-hour fifth-instar feeding trial**	**Long-term traits at pupation**
**Temperature**	11°C to 35°C fluctuating	18°C, 25°C, 32°C	Mass at fifth Consumption Mass gain Frass production	Survival Pupal mass Development time
**Diet**	1:1 (B)	1:2.5 (LP), 1:1 (B), 2.5:1 (HP)		

The experimental design included a pre-treatment period where all individuals in both populations were hatched and raised on the balanced diet through the third instar. Upon molting into the fourth instar, individuals were divided among nine treatments (three temperatures × three diets) and remained on these diets through pupation. Short-term rate measurements were taken on the first and third day of the fifth instar to calculate the 48-hour consumption, mass gain, and frass production, and long-term life-history traits were measured at pupation.

Females were kept individually in greenhouse conditions (~24°C, 60%–80% humidity, natural photocycle of 14L:10D) and given fresh collard leaves (*Brassica oleracea*) on which to oviposit. Leaves were checked daily, and eggs were removed by cutting closely around the base of each egg with dissecting scissors and placing them on a balanced diet in a large communal Petri dish inside an environmental chamber (Percival 36-VL: Percival Scientific, Perry, IA, USA), with temperatures fluctuating between 11°C and 35°C daily with a 14L:10D photoperiod until hatching. Each sibling group was kept together in the same communal dish but separated by date laid. After hatching, sibling groups were checked daily for new instars and the diet was replaced every 48 hours to maintain freshness. Upon molting into the third instar, each caterpillar was removed from the communal dish using a paintbrush and given its own unique ID number. The date of the third instar and mass were recorded, and each caterpillar was placed singly on a control 1:1 diet block inside a small, clean Petri dish. Larvae were checked daily, and diet blocks were replaced every 48 hours to maintain freshness.

Sibling groups were randomly divided across the nine treatments upon molting into the fourth instar, and the date and mass were recorded. The experiment had two components: short-term (48 hours) responses in consumption, growth, and frass production; and life-history responses in survival, development time, and mass at pupation (**Table 1**). To quantify the short-term effects, we undertook a 48-hour feeding trial with newly molted fifth instars. Each caterpillar was weighed and placed inside a clean Petri dish and given a fresh, weighed diet block (between 1,500 mg and 2,000 mg) according to their treatment. At the start of the third day of the fifth instar, the remaining uneaten diet blocks were weighed and placed inside an individually labeled, plastic ziplocked bag for later consumption analysis. After the feeding trial, caterpillars were weighed and returned to their treatments with a fresh diet to continue larval growth and development until pupation. Upon pupation, the date was recorded, and pupae were allowed to harden for 48 hours before their mass was recorded.

Consumption during the feeding trial was calculated as the diet initial dry weight minus the uneaten dry weight following a protocol modified from that in the study by Levesque et al. (43). To quantify frass production, frass generated during the feeding trial was collected and weighed inside tared microcentrifuge tubes and frozen at −80°C for later dry weight and total elemental nitrogen analyses. Fifteen pupae from different mothers from each treatment were sacrificed and frozen at −80°C to calculate dry mass ratios (five pupae) and carry out total elemental nitrogen analyses (five pupae) and total carbon analyses (five pupae). All elemental N analyses were conducted using an elemental analyzer at the North Carolina State University Environmental and Agricultural Testing Service in Raleigh, NC, USA. See the **Supplementary Material** for total elemental N analyses and more details on the drying protocols and dry mass calculations.

The remaining pupae from each treatment were allowed to eclose inside their treatment temperature in plastic cups containing a craft stick lined with a piece of damp filter paper and secured with a piece of bridal veil mesh and a rubber band. Cups were checked daily to determine the date of eclosion. Newly eclosed individuals were sexed and freeze-killed at −20°C.

### 2.3 Statistical analyses

Mass at the fifth instar and short-term consumption, mass gain, and frass production during the feeding trial were analyzed using mixed linear models in the nlme package (44) in R version 3.4.1, with diet treatment, temperature, population, and all two- and three-way interactions as fixed effects. Diet, temperature, and population were coded as factors. Because the mass at the start of the feeding trial can influence the amount consumed per individual, the mass at the start of the fifth instar was also included as a covariate for the mass gain, consumption, and frass production analyses. We also analyzed for sex differences and we found no interactions of sex with diet, temperature, or between diet and temperature; we therefore report results for the sexes combined. All analyses included mother as a random intercept. *P*-values are reported based on an analysis of variance (**Table 2**).

**Table 2:** Analysis of variance of short-term physiological metrics including mass at fifth instar, consumption, growth, and frass production during the 48-hour feeding trial.

	Mass at fifth instar		Consumption		Growth		Frass	
	F-value	*p-*value	F-value	*p-*value	F-value	*p-*value	F-value	*p-*value
**(Intercept)**	2,718.65	**<0.001**	7174.66	**<0.001**	1,825.70	**<0.001**	691.98	**<0.001**
**Population**	8.38	**0.006**	11.22	**0.002**	0.002	0.965	0.64	0.431
**Temperature**	39.61	**<0.001**	1189.45	**<0.001**	330.39	**<0.001**	82.99	**<0.001**
**Diet**	0.11	0.899	22.12	**<0.001**	49.46	**<0.001**	1.27	0.282
**Mass at fifth instar**	*NA*	*NA*	40.65	**<0.001**	6.16	**0.013**	43.37	**<0.001**
**Population:temperature**	10.80	**<0.001**	44.91	**<0.001**	3.94	**0.020**	3.59	**0.028**
**Population:diet**	2.14	0.118	28.68	**<0.001**	29.92	**<0.001**	1.17	0.311
**Temperature:diet**	0.63	0.638	2.47	**0.044**	2.23	0.065	1.42	0.227
**Population:temperature:diet**	0.53	0.715	2.38	0.051	1.92	0.107	19.72	**<0.001**

Significant *p*-values (*p* < 0.05) are highlighted in bold. Diet, temperature, and population are factors.

To assess differences in the nitrogen and carbon concentrations of the sampled pupae and frass, linear models were run using the *lm* function, with diet treatment, temperature, population, and all two- and three-way interactions as fixed effects. *P*-values are reported based on an analysis of variance (**Supplemental Table S1**).

Larval survival to pupation was analyzed as a binomial response variable using the *glmer* function in the package *lmerTest*. Three models were tested: the “full” model, with diet treatment, temperature, and population as fixed effects, their two-way and three-way interactive terms, and mother as a random effect; the “two-way” model, which contained everything in the full model except the three-way interaction term; and the “additive” model, which contained just the fixed and random effects, with no interaction terms. The two-way and additive models were individually compared with the full model to assess the impact of dropping the interactive terms, using chi-squared tests with the *anova* function. The results are reported in the text. Long-term pupal mass and development time from fourth instar to pupation were also analyzed using mixed linear models in the *nlme* package, with diet treatment, temperature, population, and all two- and three-way interactions as fixed effects. Mass at the start of the fourth instar was also included as a fixed effect for the pupal mass analysis because this was the initial mass at the start of the experimental treatments. Similarly, age at the start of the fourth instar (i.e., the number of days since hatching) was included as a fixed effect for the development time analysis to test for differences due to the developmental speeds at the start of the treatments. *P*-values are reported based on an analysis of variance (**Table 3**).

**Table 3:** Analysis of variance of the long-term life-history metrics including pupal mass and development time from hatching to pupation.

	Pupal mass		Development time	
	F-value	*p-*value	F-value	*p-*value
**(Intercept)**	3,721.48	**<0.001**	4,317.31	**<0.001**
**Population**	6.96	**0.012**	3.75	0.060
**Temperature**	43.14	**<0.001**	1068.00	**<0.001**
**Diet**	88.87	**<0.001**	78.83	**<0.001**
**Age at fourth instar**	*NA*	*NA*	1.61	0.205
**Mass at fourth instar**	56.63	**<0.001**	*NA*	*NA*
**Population: temperature**	5.21	**0.006**	0.79	0.454
**Population:diet**	38.73	**<0.001**	15.91	**<0.001**
**Temperature:diet**	3.79	**0.005**	24.30	**<0.001**
**Population:temperature:diet**	2.28	0.059	11.13	**<0.001**

Significant *p*-values (*p* < 0.05) are highlighted in bold. Diet, temperature, and population are factors.

## 3 Results

### 3.1 Short-term physiological traits

**Figure 1:**
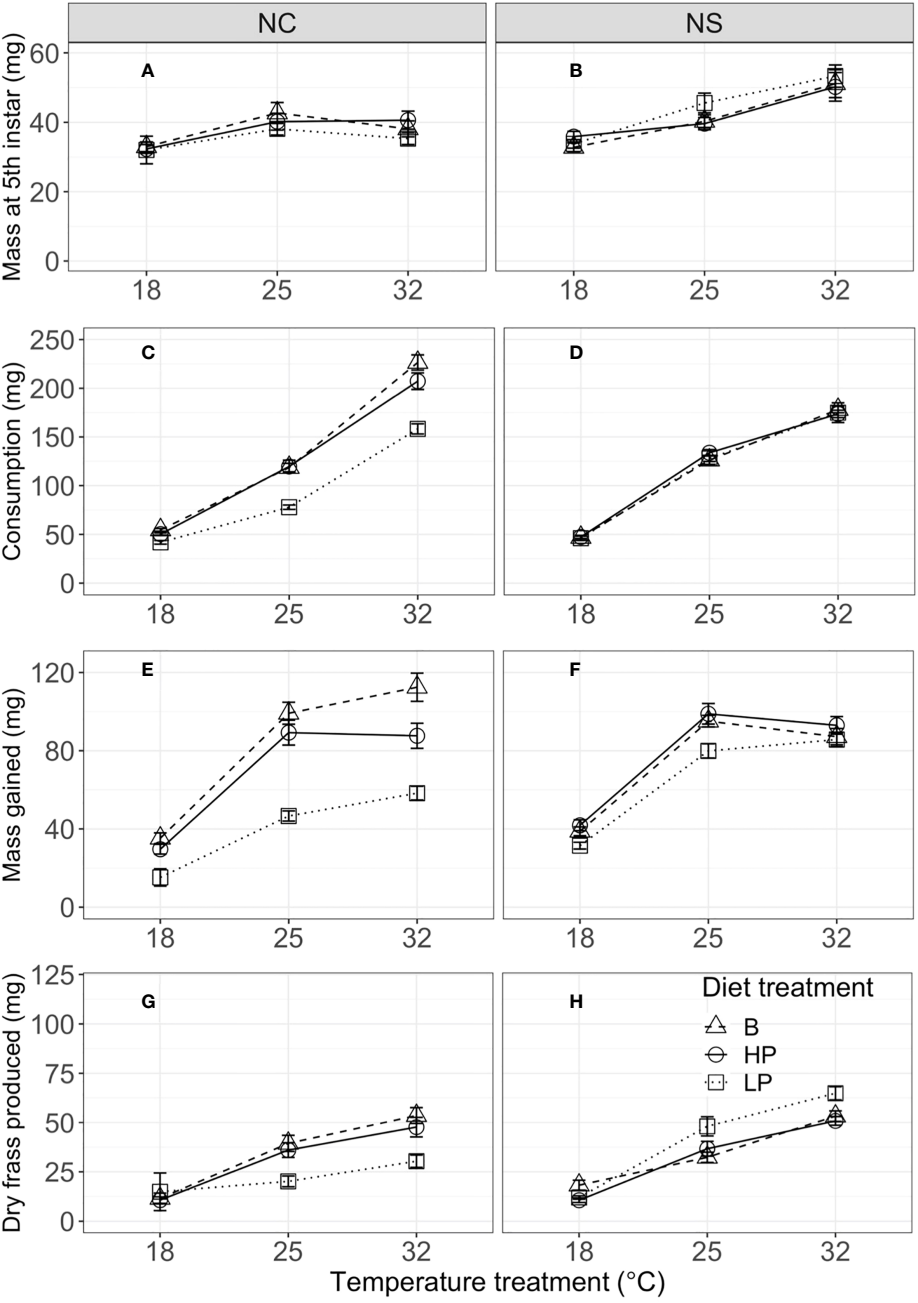
Mass at fifth instar (mg) **(A, B)**, consumption (mg) **(C, D)**, mass gain (mg) **(E, F)**, and dry frass production (mg) **(G, H)** during the 48-hour feeding trial in the fifth instar by temperature, for the North Carolina (NC, left column) and Nova Scotia (NS, right column) populations of *Pieris rapae*. Metrics are averaged over individuals within a treatment and plotted for each population with standard error bars. The linetypes represent the diet treatments.

**Figure 2:**
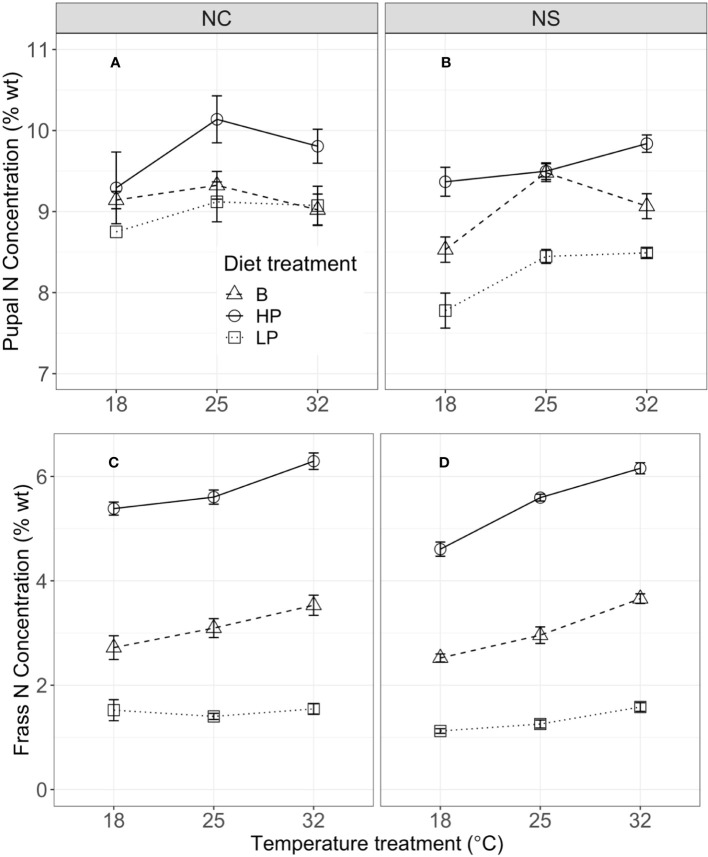
Pupal N concentration (percentage dry weight) in NC **(A)** and NS **(B)**, and frass N concentration (percentage dry weight) in North Carolina (NC) **(C)** and Nova Scotia (NS) **(D)** by temperature, for the NC (left column) and NS (right column) populations of *Pieris rapae*. Metrics are averaged over five individuals within a treatment and plotted for each population with standard error bars. The linetypes represent the diet treatments.

Mass at the fifth instar was significantly influenced by temperature, but diet did not have a significant effect (**Figures 1A, B**; **Table 2**). Short-term consumption and mass gain during the 48-hour feeding trials were significantly affected by both temperature and diet (**Table 2**). Mean consumption and mass gain increased with increasing temperature and were lower for the low P:C diet than for the balanced and high P:C diets (**Figures 1C–F**), especially for the NC population (see below). Consumption was also significantly affected by the interaction between temperature and diet (**Figures 1C, D**; **Table 2**), with greater effects of diet ratio observed at higher temperatures. Short-term frass production increased significantly with increasing temperature but was not significantly affected by the diet ratio (**Figures 1G, H**; **Table 2**). Frass N concentration (percentage dry weight) increased with increasing protein treatment, increased with temperature, and differed between populations (**Figure 2**; **Table S2**). These results suggest that short-term rates of consumption, growth, and frass production increase with increasing temperatures, and that low P:C diets can reduce these rates, at least at some temperatures.

The effects of temperature and diet on short-term physiological rates were similar in direction for the two populations, but there were some key population differences. Mass at the start of the fifth instar was significantly greater for the NS than for the NC population (**Figures 1A, B**). Consumption was significantly affected by population and the interaction of population with temperature and with diet (**Table 2**). For example, the mean consumption was higher for the NC than for the NS population, especially at higher temperatures; and diet quality had a greater effect on mean consumption in the NC than in the NS population. Similarly, for short-term mass gain, there were significant interactions of population with temperature and diet (**Table 2**; **Figures 2E, F**): both temperature and diet had a larger effect on the mean mass gain in the NC than in the NS population. There was a significant interaction between population and temperature, and a significant three-way interaction among population, temperature, and diet for short-term frass production (**Table 2**): at higher temperatures, the low P:C diet decreased the mean frass production in the NC population but increased it in the NS population (**Figures 1G, H**). Finally, the mean frass N concentration was significantly higher for NC than for NS larvae (**Figures 2C, D**). These results suggest that the effects of temperature and diet on short-term physiological rates differ substantially between these two climatically distinct populations.

### 3.2 Long-term life-history traits

**Figure 3:**
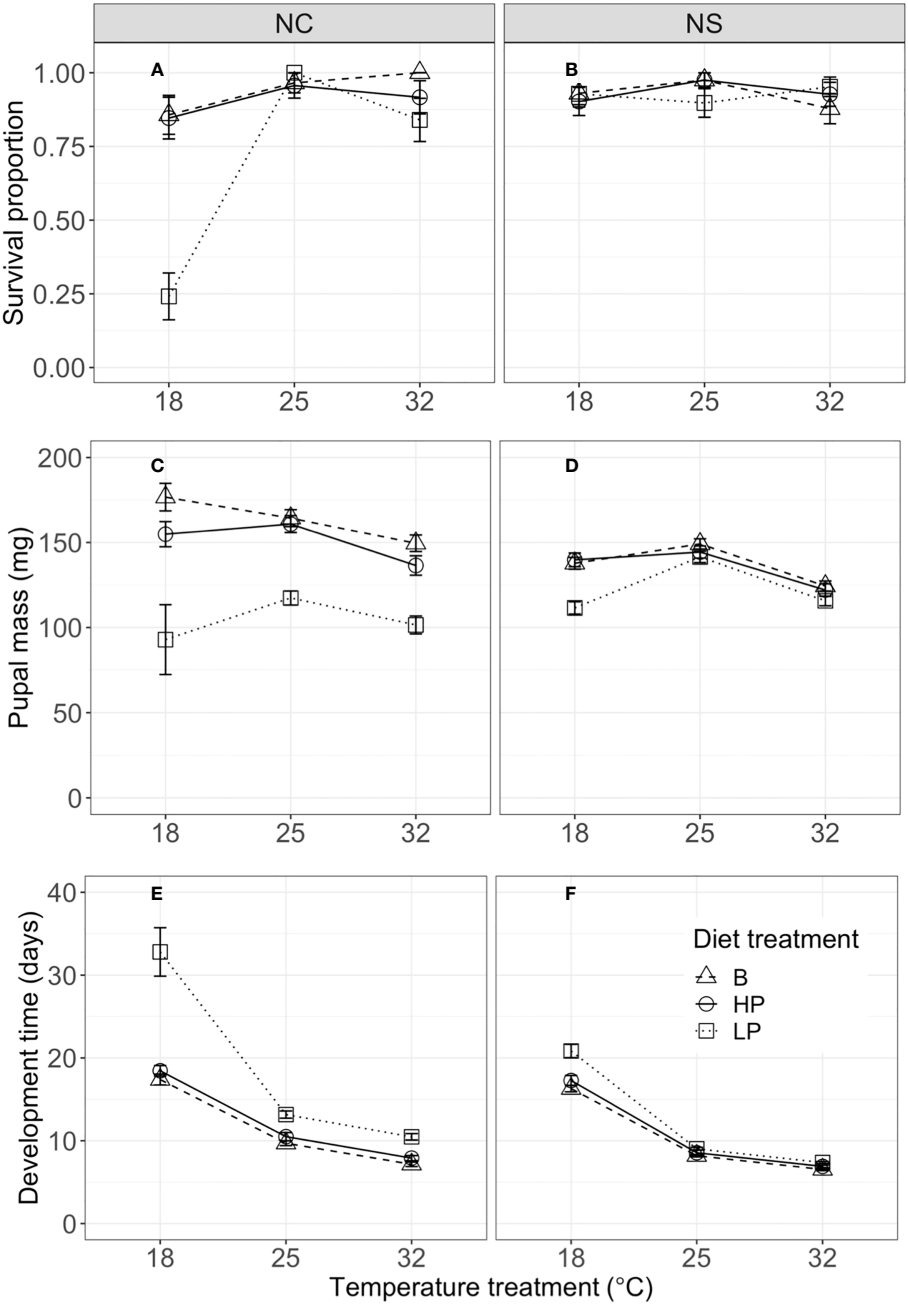
Proportion of individuals surviving to pupation **(A, B)**, pupal mass (mg) **(C, D)**, and development time from fourth instar to pupation (days) **(E, F)** by temperature, for the North Carolina (NC, left column) and Nova Scotia (NS, right column) populations of *Pieris rapae*. Metrics are averaged over individuals within a treatment and plotted for each population with standard error bars. The linetypes represent the diet treatments.

Our experiments also revealed population differences in terms of life-history traits (**Figure 3**). For survival to pupation, the full model (including all two- and three-way interactions among population, temperature, and diet) was significantly better than the additive model (without interactions) (χ ^2^_12_ = 46.212, *p* < 0.001, ΔAIC = 22.21), supporting the importance of the two-way interactions. However, the two-way model did not differ significantly from the full model (χ ^2^_4_ = 6.6819, *p* = 0.154, ΔAIC = −1.32), indicating that the three-way interaction among diet treatment, temperature, and population did not contribute substantially to the variation in survival. The NC population had a lower mean survival (84%) than the NS population (93%); this mean difference was primarily due to the low survival of the NC population (24%) when fed the low P:C diet at 18°C (**Figure 3A**). This suggests that there was a strong interaction between temperature and diet quality for survival to pupation, at least in this population.

Pupal mass was significantly affected by temperature, diet, population, and their (two-way) interactions (**Table 3**). Mean pupal masses were generally highest at 25°C, and were lower on the low P:C diet than on the balanced and high-protein diets (**Figures 3C, D**). The effects of low P:C on mean pupal mass were greater at the lowest temperatures, and were greater in the NC than in the NS population. Similarly, the pupal N concentration (percentage dry weight) was significantly affected by temperature, diet, and population (**Table S2**). The mean pupal N concentration increased with an increasing dietary P:C ratio, and was greater for the NC than for the NS population (**Figures 2A, B**). These results highlight the differing responses of pupal mass to diet quality in these two populations.

Pupal development time was significantly affected by temperature and diet, and by the interactions among temperature, diet, and population (**Table 3**). Mean development times decreased strongly with increasing temperature, and were longest for the low P:C diet, especially at the low-temperature treatment (**Figures 3E, F**). The effects of reducing dietary protein on mean development time were greater in the NC than in the NS population, especially at the lowest temperature (**Figures 3E, F**). Collectively, these results illustrate the interactive effects of temperature and diet quality on life-history traits in this species, and how these effects can differ between populations.

## 4 Discussion

### 4.1 Interactions of temperature and diet on short-term responses and life-history traits

Our results for the main effects of developmental temperate and macronutrient ratio on the short-term feeding and growth in *P. rapae* are similar to those reported for other insects (13, 21, 23), that is, increasing either temperature or dietary P:C ratio increases the short-term rates of consumption and mass gain (**Figure 1**). The rates of mass gain were similar for high-protein and balanced (1P:1C) diets but lower for low-protein diets, suggesting that dietary protein, rather than carbohydrate, is more strongly limiting for growth, which is a pattern that has been reported for many herbivorous caterpillars (12, 14). Many studies with insect herbivores, including *P. rapae*, have demonstrated increased consumption rates in response to reduced nutrient concentrations, a response which is known as compensatory feeding (13, 15); however, our results provide no evidence for compensatory feeding occurring in response to macronutrient ratio. This suggests that compensatory feeding is a response to the quantity, and not the balance, of key nutrients in this system. This could also point to post-ingestion regulation functioning as a means of nutrient imbalance management (45, 46). Notably, however, the effects of dietary macronutrients on consumption and growth vary across rearing temperature and between populations (see below). Our finding that the addition of protein to balanced diets does not improve performance in either population is also consistent with studies on diet choice in insect herbivores. For example, diet choice experiments show that most Lepidopteran larvae preferentially feed on balanced diets (1P:1C) (6), although this has not been explored for *P. rapae*.

In contrast to many studies of diet ratio, the food compositions in this study could be encountered by caterpillars in the natural environment, due to differences in agricultural fertilization practices between regions: the fertilization of crop plants increases the P:C ratio of collard leaf matter (28). Previous studies using fertilized collard greens in *P. rapae* show that consumption is decreased on non-fertilized collards in comparison with fertilized collards [(47), even at increased developmental temperatures (28)]. Hence, a significant temperature by diet interaction has ecological implications and explicitly demonstrates how nutritional conditions can influence organismal performance in relation to temperature. We chose a moderate 1P:2.5C and 2.5P:1C ratio for our experiments to produce diets that would have detectable effects without reducing survival (48, N. Morehouse, personal communication), and it is therefore very interesting that we observed such low survival (~25%) on the low-protein diet at only 18°C in the NC population.

Our results are also similar to those of studies reporting the main effects of developmental temperature and macronutrient ratio on long-term life-history traits in other insects (49–51). Increasing the temperature reduced the pupal mass and development time, whereas increasing the P:C ratio increased the pupal mass and reduced the development time (**Figure 3**). As with short-term consumption and growth, mean survival, pupal mass, and development time were similar for the balanced and high-protein diets, with significantly lower performance on the low-protein diets. Importantly, the negative consequences of a low P:C diet were greatest at the lowest rearing temperature—resulting in small pupal size and long development times under these conditions (**Figure 3**). This result illustrates how dietary quality alters the thermal reaction norms for life-history traits (37, 52, 53). For example, the slope of the thermal reaction norm for pupal size changes from negative to positive (or zero) with a decreasing P:C ratio. Several previous studies have shown that host plant quality can change the slopes of thermal reaction norms for final size in other insects, but these studies often use multiple host plant species (52) or fertilization manipulations (28). Studies with *Manduca sexta* show that secondary plant defensive compounds can alter the effects of rearing temperature on larval growth and development (54). In contrast, few studies have specifically examined the effects of P:C ratio on thermal reaction norms for life-history traits (see 21).

### 4.2 Population differences

*P. rapae* is native to Europe; it first invaded southeastern Canada (probably from Great Britain) in 1860, and by 1875 had established populations across a range of latitudes in eastern North America (39). Previous studies have demonstrated significant differences in thermal reaction norms and life-history traits among *P. rapae* populations across this latitude and climatic gradient (26). For example, common garden experiments showed that *P. rapae* from NC had faster larval growth and larger pupal mass at intermediate (i.e., 20°C–27°C) rearing temperatures than those from NS. However, population differences for the joint effects of temperature and dietary macronutrients have not been examined in this or other insect herbivores. A key finding from our study is that the subtropical (NC) population was more sensitive to the P:C ratio than the temperate (NS) population, with substantial differences in temperature by nutrient interactions found between the populations. In particular, the low P:C diet produced greater negative consequences for both short-term performance and life-history traits in the NC than in the NS population. These population differences are most striking at low developmental temperatures, at which NC individuals on the low P:C diet had much lower survival, slower growth and development, and smaller pupal mass (**Figures 1**–**3**).

Seiter and colleagues (26, 27) argued that the population differences in *P. rapae*, which have likely evolved within the last 160 years since its invasion of North America, reflect differences in seasonal climate and natural enemies along this latitudinal gradient. The growing season is much longer and mean growing season temperatures are higher in NC than in NS; the more rapid growth and larger pupal size at higher temperatures characteristic of NC *P. rapae* may allow them to complete more generations per year (four–six) than those in NS (two–three) (26). Our present results suggest that these population patterns change substantially with macronutrient balance. For example, on balanced or high-protein diets, NC individuals exhibited faster mean consumption and growth and greater pupal size than those from NS, but these population differences are reversed on a low-protein diet (**Figures 1**, **3**). Because these effects interact with temperature, nutrient balance also has large effects on the slopes of thermal reaction norms for size and development time on individuals in NC, but less so on those in NS. Plasticity in response to thermal or nutritional stress may also vary between these populations (55, 56), resulting in the differential patterns we observe.

We suggest that the differences in consumption and mass gain between populations are related to differences in their digestion ability of the low P:C diet, as quantified by frass production across treatments. In the NC population, the low-protein diet did not affect frass production during the feeding trial across temperatures, but frass production increased with increasing temperature on the balanced and high P:C diets (**Figure 1**). However, in the NS population, the low P:C diet significantly increased frass production relative to the balanced and high P:C diet, indicating some ability of the NS population to increase consumption and select for protein when fed the low-protein diet at increased developmental temperatures. Interestingly, frass N concentrations are lower across diets and temperatures in NS than in NC, suggesting an increase in N uptake in NS (**Figure 2**; **Table S2**). These results suggest that this ability is not shared by the NC population, especially not at 18°C. This difference is likely explained by differences in the climate variability and season length between populations (26).

The adaptive significance of these population differences in sensitivity to macronutrient balance is unclear. *P. rapae* populations across eastern North America utilize both domesticated *Brassica* and other wild mustards as host plants. There is a single summer growing season for domesticated *Brassica* in Nova Scotia, and multiple plantings of *Brassica* in North Carolina, primarily in spring and fall. Additionally, *Brassica* leaf N concentrations are known to increase at warmer temperatures (57, 58); this may differentiate the conditions under which NC and NS populations interact with *Brassica* sp. host plants, with NC populations being used to higher N concentrations and unable to cope with lower concentrations. The short growing season and single *P. rapae* generation in NS may select for lower sensitivity to leaf nutritional quality in this population. Alternatively, 18°C may be close to the lower thermal limit for sustainable growth for *P. rapae* larvae in NC, such that lower nutritional quality greatly reduces their growth and survival at these temperatures (52). Studies of additional populations across the geographical range of *P. rapae* would help to address these issues.

## Data availability statement

The raw data supporting the conclusions of this article will be made available by the authors, without undue reservation.

## Ethics statement

Ethical review and approval were not required for the study on animals in accordance with the local legislation and institutional requirements.

## Author contributions

JK conceived of the study and experimental design. AP conducted the final statistical analyses and figures. JK and AP revised and wrote the final manuscript. All authors contributed to the article and approved the submitted version.
