# Supplementary material for: Population divergence in nutrient-temperature interactions in Pieris rapae
Source: Front Insect Sci. 2023 Sep 11;3:1237624. doi: 10.3389/finsc.2023.1237624 (PMC10926554; doi:10.3389/finsc.2023.1237624)
Supplement: Supplementary file 1 [file DataSheet_1.pdf]

Supplemental Materials: Parker and Kingsolver, Aug 2023

Description of N analyses

A subset of diet blocks from each ratio from each population were stored at -20ºC and used for nitrogen analyses. A subset of 5 diet, pupae, and frass samples from each temperature and diet ratio treatment from each population were sent to the Environmental and Agricultural Testing Service (EATS) at North Carolina State University for total elemental carbon and nitrogen analyses by total combustion using a Perkin Elmer 2400 CHNS Analyzer. Samples were first lyophilized to a constant weight and ground so that sample particles were >4mm in diameter at UNC-CH prior to submission to EATS.

Description of the artificial diets

A meridic diet including collard powder and wheat germ was used instead of a holidic diet due to difficulty raising *P. rapae* larvae successfully on such chemically defined diets (Nathan Morehouse, University of Pittsburgh, personal communication). This meridic diet recipe was modified to produce diets containing one of three ratios of protein (casein) to digestible carbohydrates (sucrose): 13% protein with 31% digestible carbohydrates (1P:2.5C), 22% protein with 22% digestible carbohydrates (1P:1C), and 31% protein with 13% digestible carbohydrates (2.5P:1C) (% dry mass). Other constituents of the diets included 25% essential micronutrients (salts, vitamins, cholesterol) and cellulose, a nonnutritive bulking agent. The amount of cellulose added varied between diets due to differences in caloric, energy content between casein and sucrose.

Description of dry consumption, frass production protocol and calculations

A subset of control diet blocks from each diet ratio was placed in each of the three test temperatures to assess water loss during the 48 feeding trials. These diet blocks were weighed before and after the trial, and dried to a constant mass in a 50°C drying oven for 72 hours. Initial dry weight was estimated by incorporating the initial fresh weight of the diet block in a line-of-fit equation computed from a scatterplot regression of the dry weight (x) against fresh weight (y) of control diet blocks (15 leaves per treatment and cohort).

Similarly, a subset of 5 frass samples per treatment were dried after the completion of the experiment to a constant mass at 50ºC for 72 hours to calculate frass production during the feeding trial per treatment. Here the dry weight of each frass sample was divided by the wet weight for each frass sample, and this ratio of dry over wet weight was then averaged for 5 frass samples for each treatment. The average ratio was then multiplied by the wet mass for all frass in each treatment to obtain a dry weight estimation for all frass in the experiment. To estimate dry weight of pupae, a subset of 5 of these frozen pupae were dried at 50°C for 72 hours until they reached a constant mass using the same protocol used to calculate frass dry weights.

Table S1. Composition of the low protein (LP), balanced (B), and high protein (HP) artificial diets used in the experiments. Modified from Troetschler et al., (1985).

**Ingredient**	**1:2.5 (LP)**	**1:1 (B)**	**2.5:1 (HP)**
Agar (g)	30	30	30
Wheat germ (g)	48	48	48
Casein (g)	34	82	130
Cellulose (g)	89	65	42
Sucrose (g)	51	27	2
Wesson's salt mix (g)	18	18	18
Torula yeast (g)	15	15	15
Cholesterol (g)	7.2	7.2	7.2
Collard leaf powder (g)	30	30	30
Sorbic acid (g)	3	3	3

Methyl paraben (g)	1.5	1.5	1.5
Ascorbic acid (g)	6	6	6
Vanderzant's vitamin mix (g)	21	21	21
Streptomycin sulfate (g)	0.35	0.35	0.35
Water (boiling) (ml)	700	700	700
Water (cool) (ml)	600	600	600
Linseed oil (ml)	10	10	10

Table S2: ANOVA of the pupal and frass N and C concentration analyses. Significant *p*-values ($p < 0.05$) are highlighted in bold. Diet, temperature, and population are factors.

	**Pupal N concentration**		**Frass N concentration**		**Pupal C concentration**		**Frass C concentration**	
	F-value	*p*-value	F-value	*p*-value	F-value	*p*-value	F-value	*p*-value
**Population**	18.10	**<0.001**	14.78	**<0.001**	25.09	**<0.001**	10.89	**0.002**
**Temperature**	12.29	**<0.001**	1498.61	**<0.001**	1.51	0.228	124.44	**<0.001**
**Diet**	37.02	**<0.001**	49.93	**<0.001**	30.41	**<0.001**	105.06	**<0.001**
**Pop:Temp**	1.02	0.366	3.66	**0.031**	3.66	**0.031**	5.99	**0.004**
**Pop:Diet**	3.25	0.045	0.56	0.576	3.08	0.053	7.47	**0.001**
**Temp:Diet**	0.64	0.637	6.80	**<0.001**	1.80	0.140	5.92	**<0.001**
**Pop:Temp:Diet**	1.70	0.159	0.86	0.489	1.13	0.351	1.05	0.387

Supplemental Figure 1

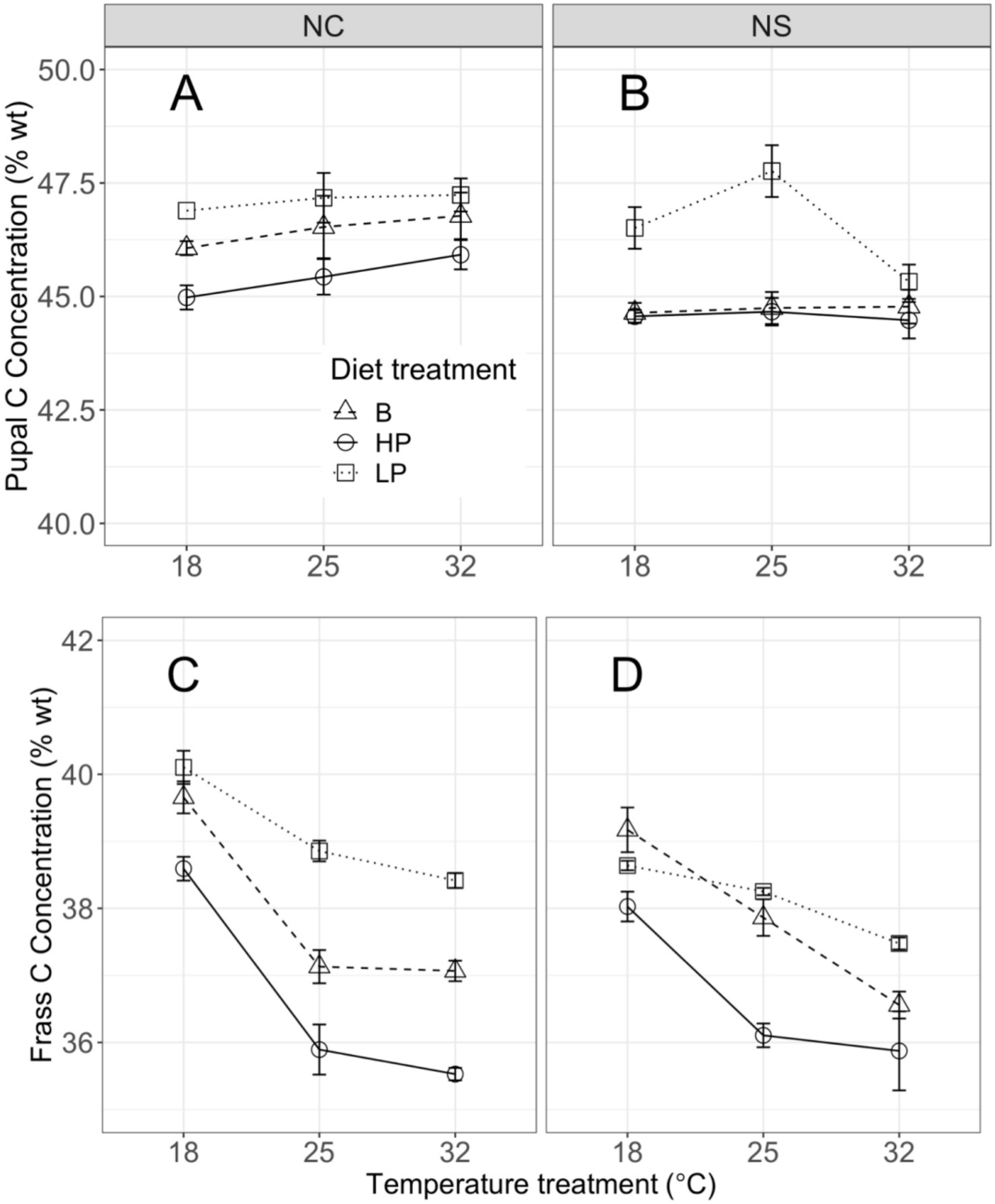

SF1. Pupal C concertation (% dry wt) in NC (A) and NS (B), and frass C concentration (% dry wt) in NC (C) and NS (D) by temperature. Metrics are averaged over 5 individuals within a treatment and plotted per population with standard error bars. Linetypes represent diet treatment.
